# Evaluation of the Psychometric Properties of the Scale A-ONE: An Italian Cross-Sectional Study

**DOI:** 10.1155/2021/8874953

**Published:** 2021-03-17

**Authors:** Margherita Cerone, Marco Tofani, Giovanni Fabbrini, Giulia Marcellini, Anna Berardi, Claudia Conti, Rita De Santis, Donatella Valente, Giovanni Galeoto

**Affiliations:** ^1^San Giovanni Battista-Cavalieri di Malta, Rome, Italy; ^2^Neurorehabilitation Unit, Department of Intensive Neurorehabilitation and Robotics, Bambino Gesù Children's Hospital, Italy; ^3^Department of Human Neurosciences, Sapienza University of Rome, Italy; ^4^IRCCS Neuromed Pozzilli, Italy; ^5^Sapienza University of Rome, Rome, Italy; ^6^Department of Anatomical, Histological, Forensic and Orthopedic Sciences, “Sapienza” University of Rome, Italy

## Abstract

**Purpose:**

This study is aimed at validating the A-ONE scale in an Italian population with Central Nervous System (CNS) dysfunction. *Material and Methods*. Between May and November 2018, people aged between 60 and 90 with CNS dysfunction were recruited in a hospital in Rome, Italy. Patients were observed and evaluated during the activities of daily living. Internal consistency and reliability were evaluated with Cronbach's coefficient alpha and intraclass correlation coefficient, respectively. As measured with Pearson's correlation coefficient, the validity was examined comparing results of the A-ONE with the Barthel index. Responsiveness was evaluated 30 days after the first administration.

**Results:**

A total of 70 people having a diagnosis of neurological disorders were evaluated. The internal consistency showed Cronbach's coefficient alpha ranging from 0.634 to 0.959. The measurement of reliability varied from 0.984 to 0.997 for intrarater and from 0.979 to 0.998 for interrater. Pearson's correlation coefficient between the A-ONE and the Barthel index and the responsiveness showed statistically significant values (*p* < 0.01).

**Conclusions:**

The present study provides preliminary evidence of reliability, validity, and responsiveness of the A-ONE when using elderly people with CNS dysfunction.

## 1. Introduction

Occupational therapy is an integral part of the rehabilitation process of people with brain injuries. Central Nervous System (CNS) diseases are the dysfunction of any brain component (including the cerebral hemispheres, the diencephalon, the brain stem, and the cerebellum) or the spinal cord. Several causes of these dysfunctions result in neurobehavioral deficits, including vascular disorders, metabolic disorders, head trauma, infections, toxins, brain tumors, and degeneration of the nervous system [1]. The dysfunction of the CNS is evident in patients with various diagnoses. In Italy, diseases of the nervous system that require a specialist neurologist intervention show an incidence of 7.5% per year and a prevalence of 30% [2].

Regarding patient evaluation, in rehabilitation, there are several rating scales. To test which skills and abilities the individual has lost or maintained, the occupational therapist takes its point of reference, the activities of daily living (ADL) [3–5]. Traditionally, this type of evaluation is used exclusively to indicate the level of independence and need for assistance. Rather, it is possible using the ADL-focused Occupation-based Neurobehavioral Evaluation (A-ONE) [6] to grasp the reasons for lack of independence or understand how or why this dysfunction interferes with daily performance.

The A-ONE was published for the first time in 1990 by occupational therapist Guðrún Árnadóttir. She aimed to create a tool based on an occupation to simultaneously evaluate the performance in ADL and the impact of the neurobehavioral impairment, which limits the performance in the natural environment [7–9].

This tool is designed to be used with people aged above 16, with neurological and neurocognitive disorders due to a CNS dysfunction (e.g., stroke, head trauma, Alzheimer's disease, and Parkinson's disease), and is specific to occupational therapists.

The usage of the A-ONE requires the observation of the performance during ADL. The A-ONE is composed of an ADL scale, termed the Functional Independence scale, and a Neurobehavioral Impairment scale, composed of two subscales, the Neurobehavioral Specific Impairment subscale and the Neurobehavioral Pervasive Impairment subscale [10]. A certification course lasting five days is required for using the tool effectively. Training courses have been organized all over the world since 2013, including in Japan, Iceland, Holland, the USA, Denmark, Canada, Italy, Korea, and Norway (http://www.a-one.is). Other than the original version, the A-ONE was translated and validated for Japanese [11] and Korean [12] population. Psychometric property studies reveal good validity and reliability [10–12].

The study is aimed at testing the scale's psychometric properties for the Italian context, namely, the internal consistency, reliability, validity, and responsiveness. Therefore, this study's expected outcome is to provide a useful tool to most occupational therapists who will evaluate patients with brain dysfunction to verify functional independence concerning neurobehavioral impairments.

## 2. Material and Methods

The study was conducted by a research group composed of medical doctors and rehabilitation professionals of the Sapienza University of Rome, Tor Vergata University of Rome, and from the Rehabilitation and Outcome Measure Assessment (ROMA) Association. In the last few years, ROMA has dealt with the validation of many outcome measures in Italy [4, 5, 13–24].

### 2.1. Description of the Sample

The people were recruited from May to November 2018 at the rehabilitation center at the San Giovanni Battista–Cavalieri di Malta, Rome. Sample size dimension was defined confronting other validity studies: the validation study of the Japanese version of the A-ONE (sample 65) [11] and the validity study for cerebrovascular accident (sample 45) [25]. Therefore, the research group was defined as a minimum sample size of 65 people. The A-ONE was created to guide the occupational therapist in the intervention planning. However, Arnadottir and Fisher (2008) verify that one of the A-ONE subscales (ADL scales) could also be an outcome measure in terms of the occupational therapist intervention's efficacy. In our clinical practice, the A-ONE was used as a guiding tool for OT practice, and an outcome measure. To be admitted to the study, patients had to meet the following inclusion criteria: diagnosis of a neurological disorder of the CNS, being 60 to 90 years old, and a signature for the informed consent. All people who had other medical conditions or comorbidities (e.g., hip prosthetics) were excluded. Before the evaluation, the patient was provided information regarding the study. Once the patient clarified any doubts about objectives and procedures, they were asked to sign an informed consent [22, 26].

### 2.2. Measurements

A-ONE: the A-ONE is commonly used for adults that have acquired CNS dysfunction. A-ONE is composed of two scales: the Functional Independence scale and the Neurobehavioral Impairment scale.

The Functional Independence scale consists of 20 ADL items and two communication items. It is a 5-category ordinal rating scale ranging from 0 to 4 used to score the observed level of assistance needed to overcome the impact of impairment on ADL performance (0 = full assistance needed, 1 = minimum to considerable physical assistance needed, 2 = verbal assistance needed, 3 = supervision needed, and 4 = independent) [8].

The Neurobehavioral Impairment scale is used to assess the extent to which the consequences of neurobehavioral alterations affect performance in daily living activities. The Neurobehavioral Impairment scale contains two subscales, the Neurobehavioral Specific Impairment subscale comprised of 46 rating scale items and the Neurobehavioral Pervasive Impairment subscale comprised of 31 dichotomous items [6]. Most of the specific impairment items are independently rated more than once in connection with the performance of different ADL tasks (e.g., motor apraxia-dressing, motor apraxia-grooming and hygiene, motor apraxia-transfers, and mobility, and motor apraxia-feeding). The Neurobehavioral Pervasive Impairment subscale items are rated only once based on an observed error in at least one ADL task. The 5 ADL tasks observed (dressing, grooming and hygiene, transfers and mobility, feeding, and communication) are referred to as ADL domains. Persons are scored based on assistance to overcome the occupational errors during ADL task performance [7].

The Barthel's index [26] provides a score which is indicative of the capacity of the patient for autonomy: feeding, bathing, care of appearance, dressing, using the toilet, moving from wheelchair to bed and vice versa, walk on surfaces such as the floor, climb up and down the stairs, and control the bowel and bladder. The scores assigned to each function can be 15, 10, and 5. The maximum score is assigned only if the patient performs the task completely independently, without the presence of personal assistance. Otherwise, a value down to zero is assigned. The maximum score for each function is assigned to have a total score of 100; such a score would indicate complete independence in daily living activities (Castiglia et al., 2017).

### 2.3. Reliability and Validity

The patients were observed and evaluated during the ADL in the morning by two certified occupational therapists who have completed the A-ONE course.

For the intrarater reliability, the patient was evaluated twice by the same rater, one day after the first evaluation. A short evaluation time (1 day) was chosen because the study setting included acute and postacute units. For interrater reliability, the two OT evaluated the same patient at the same time. Raters were blinded to each other's results. To analyze reliability, the intraclass correlation coefficient (ICC) was used. The scale is considered to be reliable if the ICC is higher than 0.70. The A-ONE scale's internal consistency was assessed through Cronbach's coefficient alpha, whose value can vary from 0 to 1; it is considered significant when greater than 0.70 [27, 28].

To compare the A-ONE scores, Barthel's index was used as a gold standard for construct validity. Pearson's correlation coefficient was calculated to analyze validity, which measures the degree of association between two variables, resulting in values between -1 and 1 [29].

### 2.4. Responsiveness

To measure A-ONE responsiveness in a subpopulation of the sample, the Wilcoxon rank test (*p* < 0.05) was used in patients who performed an occupational therapy intervention. The range was calculated based on hospitalization timing during an intensive rehabilitation program, so responsiveness was evaluated at the discharge after 30 days from the first assessment.

## 3. Results

A total of 70 people having a diagnosis of neurological disorders were evaluated; the mean age is 74.66 (SD 7.88) years (range 60-89). The main characteristics of the sample are described in Table 1.

### 3.1. Reliability

The internal consistency of the A-ONE analyzed through Cronbach's alpha showed good values, ranging from 0.634 (in the domain of communication) to 0.930 (in the domain of transfers and mobility), while values for the total score were 0.959.  Table 2 summarizes data for internal consistency, while Table 3 reports item-total correlation.

All patients enrolled in the study were evaluated twice by the same rater one day after the first evaluation (intrarater reliability). As shown in Table 4, the ICC of all domains varied from 0.984 to 0.997. Furthermore, interrater reliability showed values higher than 0.70, which varied from 0.979 to 0.998, indicating a high agreement between the two evaluations. Results are summarized in Table 5.

### 3.2. Validity

The construct validity was evaluated using Pearson's correlation coefficient.  Table 6 shows the results of the correlation between A-ONE and the Barthel index.

### 3.3. Responsiveness

Responsiveness was measured in 36 patients, compared to 70 of the population enrolled in the study. This is because only 36 patients received an occupational therapy intervention.  Table 7 reports the main results.

## 4. Discussion

The purpose of this study was to evaluate the psychometric properties of the A-ONE in an Italian population with CNS dysfunction. Aforementioned, the A-ONE is divided into two subscales: the Functional Independence scale and the Neurobehavioral Impairment scale [25]. The Neurobehavioral Impairment scale's original purpose was to determine the nature of problems that interfere with ADL task performance. The goal was to identify the type and severity of neurobehavioral impairment limiting the individual's independence in ADL tasks (e.g., motor apraxia, unilateral body neglect, and attention) and to gather the information that could be used to guide occupational therapy interventions [7]. Therefore, the present study reports only psychometric properties of the Functional Independence scale.

Cronbach's coefficient alpha of the Functional Independence subscale ranging from 0.634 to 0.930 was consistent with the original study (*α* = 0.75–0.79). For reliability analysis, our result on interrater reliability reports very high values (>0.95), in line with those reported by Arnadottir and colleagues (2008) (ICC = 0.98). However, to the best of our knowledge, no studies report values on intrarater reliability. Our study presents the first values of intrarater reliability and highlights the stability of the scale between raters.

Construct validity values reveal a good relationship with the Barthel index, except for the communication domain (0.261). The present result can be explained because the Barthel index does not measure communication at all.

Responsiveness was measured in a subsample population because the whole population has not received an occupational therapy intervention. Our results show that the A-ONE scale can register improvement after rehabilitation and can be used in current clinical practice.

Despite these encouraging results, the present study has some limitations. First of all, the lack of validation study in other countries does not compare some psychometric properties in different contexts. Second, the relatively small sample size does not allow to understand differences between different population. Further study should explore a bigger sample with different CNS diseases.

## 5. Conclusion

The present investigation reveals A-ONE as a valid and reliable tool when using with people with CNS dysfunction. Now, Italian occupational therapists can measure with more confidence activities of daily living and the performance of their clients.

## Figures and Tables

**Table 1:** Demographic characteristics of the 70 patients enrolled in the study.

Variable	Population = 70
Gender female n° (%)	46 (66)
Age mean ± SD (range)	74.66 ± 7.88 (60-89)
Diagnosis n° (%)	
Stroke	51 (73)
Head trauma	6 (9)
Parkinson's disease	13 (19)
Marital status n° (%)	
Married	37 (53)
Divorced	4 (6)
Unmarried	5 (7)
Separate	5 (7)
Widower	19 (27)
Employment status n° (%)	
Employed	12 (17)
Unemployed	19 (27)
Retired	39 (56)
Neurobehavioral pervasive impairment^∗^ n° (%)	
Lability	2 (2.9)
Euphoria	1 (1.4)
Apathy	12 (17.1)
Depression	14 (20)
Frustration	8 (11.4)
Restlessness	2 (2.9)
Concrete thinking	5 (7.1)
Decreased insight	7 (10)
Impaired judgment	9 (12.9)
Confusion	10 (14.3)
Impaired alertness	2 (2.9)
Impaired attention	3 (4.3)
Distractibility	1 (1.4)
Impaired initiative	17 (24.3)
Impaired motivation	20 (28.6)
Performance latency	20 (28.6)
Absent mindedness	2 (2.9)
Short-term memory	6 (8.6)
Long-term memory	5 (7.1)
Disorientation	9 (12.9)

**Table 2:** Internal consistency reliability for the different domains.

	N° of items	Cronbach's alpha	Cronbach's alpha based on standardized elements
Dressing	5	0.926	0.927
Grooming and hygiene	6	0.881	0.884
Transfers and mobility	5	0.930	0.930
Feeding	4	0.774	0.851
Communication	2	0.634	0.663
Total	22	0.959	0.957

**Table 3:** Cronbach's alpha of the scale A-ONE (items 1-22) eliminating individual items.

	Medium scale if it deleted the item	Variance scale if it deleted the item	Correlation item-total correct	Cronbach's alpha if it deleted the item
Item1-D	47.04	430.389	0.773	0.956
Item2-D	47.70	426.851	0.826	0.956
Item3-D	47.86	428.936	0.785	0.956
Item4-D	47.76	424.592	0.848	0.955
Item5-D	47.21	429.852	0.738	0.957
Item6-G/H	46.54	435.962	0.739	0.957
Item7-G/H	46.44	439.497	0.658	0.958
Item8-G/H	46.84	427.149	0.776	0.956
Item9-G/H	48.69	441.755	0.564	0.959
Item10-G/H	48.00	413.681	0.886	0.955
Item11-G/H	48.81	441.603	0.701	0.957
Item12-T/M	47.11	427.668	0.784	0.956
Item13-T/M	47.40	421.142	0.895	0.955
Item14-T/M	46.71	429.135	0.749	0.956
Item15-T/M	47.90	410.932	0.885	0.955
Item16-T/M	48.63	435.280	0.712	0.957
Item17-F	45.83	455.883	0.536	0.959
Item18-F	45.83	456.724	0.514	0.959
Item19-F	46.06	448.750	0.620	0.958
Item20-F	47.83	430.173	0.634	0.958
Item21-C	45.64	469.131	0.314	0.960
Item22-C	46.16	464.656	0.330	0.960

D = dressing; G/H = grooming and hygiene; T/M = transfers and mobility; F = feeding; C = communication.

**Table 4:** Test-retest reliability intraoperator (after 24 hours) for different domains.

	Test (mean ± SD)	Retest (mean ± SD)	ICC	IC 95%		*p*
Dressing	9.6 ± 6.3	9.71 ± 6.2	0.994^c^	0.990	0.996	<0.001
Grooming and hygiene	11.24 ± 6.7	11.46 ± 6.6	0.997^c^	0.995	0.998	<0.001
Transfers and mobility	9.39 ± 6.7	9.51 ± 6.5	0.996^c^	0.994	0.998	<0.001
Feeding	12.17 ± 3.6	12.36 ± 3.6	0.992^c^	0.987	0.995	<0.001
Communication	7.06 ± 1.2	7.13 ± 1.2	0.984^c^	0.975	0.990	<0.001

**Table 5:** Test-retest reliability interoperator (two operators at the same time) for different domains.

	Test (mean ± SD)	Retest (mean ± SD)	ICC	IC 95%		*p*
Dressing	9.57 ± 6.3	9.44 ± 6.2	0.996^c^	0.994	0.998	0.001
Grooming and hygiene	11.24 ± 6.7	11.14 ± 6.7	0.998^c^	0.996	0.998	0.001
Transfers and mobility	9.39 ± 6.7	9.39 ± 6.7	0.996^c^	0.994	0.998	0.001
Feeding	12.17 ± 3.6	12.17 ± 3.7	0.989^c^	0.982	0.993	0.001
Communication	7.06 ± 1.2	7.10 ± 1.3	0.979^c^	0.967	0.987	0.001

**Table 6:** Pearson's correlation coefficient for the totals of each A-ONE domain with respect to the total of Barthel's index.

		Dressing	Grooming and hygiene	Transfers and mobility	Feeding	Communication
Barthel	Pearson's correlation	0.905^∗∗^	0.889^∗∗^	0.920^∗∗^	0.671^∗∗^	0.261^∗^

^∗∗^Correlation is significant at the 0.01 level (2-tailed). ^∗^Correlation is significant at the 0.05 level (2-tailed).

**Table 7:** Responsiveness of A-ONE measured after 30 days of occupational therapy intervention.

	T0 Mean ± SD	T1 Mean ± SD	*Z*	Sign.
Dressing	6.17 ± 2.26	12.53 ± 4.30	-5.093^b^	<0.001
Grooming and hygiene	8 ± 4.16	13.44 ± 3.56	-5.171^b^	<0.001
Transfers and mobility	5.78 ± 3.68	11.53 ± 4.54	-4.943^b^	<0.001
Feeding	11.67 ± 2.74	13.22 ± 1.97	-4.422^b^	<0.001
Communication	7.11 ± 1.19	7.61 ± 0.60	-3.216^b^	0.001

## Data Availability

The research data used to support the findings of this study are included within the article.

## Additional Points

*Implication for Rehabilitation*. (i) The A-ONE assesses the independence in daily life activities and the impact of neurobehavioral impairment on performance (no scale was developed to evaluate impairment impact on performance). (ii) It can be used for many neurological disorders in patients aged above 16. (iii) It is an occupation-based evaluation tool specific for occupational therapists. (iv) It helps in clinical reasoning, decision-making, and selection of intervention
